# Identification of Markers Predicting Clinical Course in Patients with IgG4-Related Ophthalmic Disease by Unbiased Clustering Analysis

**DOI:** 10.3390/jcm9124084

**Published:** 2020-12-17

**Authors:** Kinya Tsubota, Yoshihiko Usui, Rey Nemoto, Hiroshi Goto

**Affiliations:** Department of Ophthalmology, Tokyo Medical University Hospital, Tokyo 160-0023, Japan; usuyoshi@gmail.com (Y.U.); nemotorey@gmail.com (R.N.); goto1115@tokyo-med.ac.jp (H.G.)

**Keywords:** IgG4-related ophthalmic disease, unbiased cluster analysis, extraocular muscle enlargement, decreased best corrected visual acuity, biomarker

## Abstract

Purpose: To describe the clinical features of patients with immunoglobulin G4 (IgG4)-related ophthalmic disease (IgG4-ROD) grouped by unbiased cluster analysis using peripheral blood test data and to find novel biomarkers for predicting clinical features. Methods: One hundred and seven patients diagnosed with IgG4-ROD were divided into four groups by unsupervised hierarchical cluster analysis using peripheral blood test data. The clinical features of the four groups were compared and novel markers for prediction of clinical course were explored. Results: Unbiased cluster analysis divided patients into four groups. Group B had a significantly higher frequency of extraocular muscle enlargement (*p* < 0.001). The frequency of patients with decreased best corrected visual acuity (BCVA) was significantly higher in group D (*p* = 0.002). Receiver operating characteristic (ROC) curves for the prediction of extraocular muscle enlargement and worsened BCVA using a panel consisting of important blood test data identified by machine learning yielded areas under the curve of 0.78 and 0.86, respectively. Clinical features were compared between patients divided into two groups by the cutoff serum IgE or IgG4 level obtained from ROC curves. Patients with serum IgE above 425 IU/mL had a higher frequency of extraocular muscle enlargement (25% versus 6%, *p* = 0.004). Patients with serum IgG4 above 712 mg/dL had a higher frequency of decreased BCVA (37% versus 5%, *p* ≤ 0.001). Conclusion: Unsupervised hierarchical clustering analysis using routine blood test data differentiates four distinct clinical phenotypes of IgG4-ROD, which suggest differences in pathophysiologic mechanisms. High serum IgG4 is a potential predictor of worsened BCVA, and high serum IgE is a potential predictor of extraocular muscle enlargement in IgG4-ROD patients.

## 1. Introduction

Immunoglobulin G4-related disease (IgG4-RD) is a disease entity of unknown cause. The disease is characterized by the formation of mass lesions resembling tumor, thickening of various tissues, infiltration of immunoglobulin G4 (IgG4)-positive cells, and high serum IgG4 [1,2]. Although the concept of IgG4-RD as a systemic disease has been recognized relatively recently, the diseases that were clinically diagnosed as autoimmune pancreatitis in gastroenterology and Mikulicz disease in ophthalmology in the past have been revealed to be lgG4-RD [1,2]. Thereafter, the presence of similar masses or thickened lesions in various organs such as the hepatobiliary system, lung, kidney, prostate, and thyroid in addition to the pancreas, lacrimal gland (LG), and salivary glands (SG) was revealed, and comprehensive diagnostic criteria for IgG4-RD were reported in 2012 [3,4]. Moreover, other than LG and SG, some patients with IgG4-RD have enlargement of ocular adnexal tissues such as extraocular muscles, trigeminal nerve, and orbital fat. Ocular involvement is one of the most common factors of disability caused by IgG4-RD, and has been reported to affect nearly 12–65% of patients with IgG4-RD [5,6,7,8]. All the ocular manifestations are collectively called IgG4-related ophthalmic disease (IgG4-ROD), and the diagnostic criteria for IgG4-ROD were published in 2015 [9]. Although the pathogenesis of IgG4-RD and IgG4-ROD is not completely understood, an infectious or an autoimmune or autoinflammatory etiology has been proposed [10,11,12,13]. Ophthalmologists have been aware that the clinical presentation and symptoms of IgG4-ROD vary. For example, some patients improve upon treatment with systemic steroids with no recurrence, while others do not respond to systemic steroids; some patients have more severe disease and systemic involvement, whereas others do not. Therefore, the fact that IgG4-ROD has diverse manifestations has driven interest in using unbiased approaches to better phenotype these patients in terms of essential clinical and laboratory features. Recently, elevations of serum immunoglobulin G2 and soluble interleukin-2 receptor (sIL-2R) in IgG4-RD have been reported [14,15]. However, biomarkers were identified based on the clinical features of IgG4-ROD.

Cluster analysis refers to statistical methods that attempt to discriminate relatively homogeneous groups of individuals based on selected characteristics. Recently, cluster analysis has been used to identify phenotypic groups within a disease for various diseases [6,16,17]. To date, only one study of IgG4-RD including IgG4-ROD using cluster analysis has been reported [6]. This study showed that the total dose of systemic steroids used in maintenance therapy was significantly greater in the cluster of patients with hypergammaglobulinemia, elevated serum IgG4 levels, and hypocomplementemia than in the cluster of patients with older onset and relatively low peripheral eosinophil count. Moreover, the cluster of patients with elder onset and relatively low peripheral eosinophil count had a low rate of relapse and often were able to discontinue steroids [6]. To more comprehensively analyze IgG4-ROD, we used an unsupervised modeling method to study a relatively large number of conventional laboratory blood test data, aiming to identify clusters of individuals with IgG4-ROD and to evaluate the degree of phenotypic heterogeneity.

As IgG4-ROD is a heterogeneous disease with diverse clinical symptoms, the goal of IgG4-ROD classification is to improve prediction of the phenotype of an individual patient, so as to aid ophthalmologists in making a decision on treatment strategy (such as whether to prescribe systemic steroid therapy), ultimately achieving personalized or stratified care for IgG4-ROD.

## 2. Materials and Methods

We conducted a retrospective, non-interventional, single-institutional observational study. This research was conducted in compliance with the Helsinki principles. Ethical approval was obtained from the Medical Ethical Research Committee in Tokyo Medical University Hospital (Ethic code: 2016-150). All patients signed an informed consent form before participation.

### 2.1. Patients

We reviewed the medical records at the Department of Ophthalmology, Tokyo Medical University Hospital between July 2008 and March 2020, and identified 152 patients with IgG4-ROD. Among these patients, 45 were excluded because of no peripheral blood test data for soluble IL-2 receptor (sIL-2R) and beta2-microglobulin (β2MG) (25 patients), prednisolone (PSL) treatment initiated before presentation to our hospital (17 patients), and no data of gene rearrangement (3 patients). The remaining 107 patients with IgG4-ROD were included in this analysis. The subjects (55 males and 52 females) were all Asian adults with an average onset age of 57.3 ± 14.5 years. All patients underwent magnetic resonance imaging (MRI) or computer tomography (CT) to evaluate the location of lesion. Lesion sites were evaluated by two ophthalmology specialists using MRI or CT images. Peripheral blood tests were performed at the first visit to our hospital. Biopsy was performed on all patients who had a palpable mass and gave informed consent to undergo biopsy. Gene rearrangement and flow cytometry were performed in all patients who underwent biopsy. The diagnosis of IgG4-ROD was determined based on the diagnostic criteria for IgG4-ROD [9]. The following clinical data were extracted from the medical records of each patient: sex, gender, age, follow-up period, absence or presence of biopsy, type of IgG4-ROD (definite, probable, and possible), enlargement of LG, bilateral or unilateral LG enlargement, enlargement of SG, lesion other than LG and SG, enlargement of trigeminal nerve, enlargement of extraocular muscle, presence of orbital mass, presence of orbital diffuse lesion, swelling of eye lid, decrease of best corrected visual acuity (BCVA), optic neuropathy, visual field defect, diplopia, dry eye, use of systemic PSL, local injection of triamcinolone acetonide (TA), frequency of recurrences, number of recurrence, and results of peripheral blood tests. Worsened BCVA was defined as acute worsening of BCVA within 1 month. Recurrence was defined as re-enlargement of lesion shown on MRI or CT.

### 2.2. Clustering Analysis

Unsupervised hierarchical clustering analysis was performed using the following continuous variables: peripheral white blood cell (WBC) count, percent lymphocyte (Lym), percent eosinocyte (Eo), peripheral platelet (PLT) count, serum total protein (TP), aspartate aminotransferase (AST), alanine aminotransferase (ALT), γ-glutamyltransferase (γ-GTP), lactate dehydrogenase (LDH), alkaline phosphatase (ALP), total bilirubin (T-BIL), blood urea nitrogen (BUN), creatinine (Cre), C-reactive protein (CRP), immunoglobulin G (IgG), immunoglobulin A (IgA), immunoglobulin E (IgE), β2MG, sIL-2R, and serum IgG4. Analysis was performed using statistical software (BellCurve for Excel, Social Survey Research Information Co. Ltd., Tokyo, Japan; JMP, SAS Institute, Ltd., Tokyo, Japan). We divided the patients into four groups by unbiased clustering analysis using Ward’s method [18,19]. We then compared the four groups divided by unbiased clustering analysis for the following variables: sex, gender, age, follow-up period, absence or presence of biopsy, type of IgG4-ROD (definite, probable, and possible), enlargement of LG, bilateral or unilateral of enlargement of LG, enlargement of SG, lesion other than LG and SG, enlargement of trigeminal nerve, enlargement of extraocular muscles, presence of orbital mass, presence of orbital diffuse lesion, swelling of eyelid, worsening of BCVA, optic neuropathy, visual field defect, diplopia, dry eye, use of systemic PSL, local injection of TA, frequency of recurrence, and number of recurrence.

### 2.3. Receiver Operating Characteristic (ROC) Curves

ROC curves were generated to predict extraocular muscle enlargement, worsened BCVA, and lesion above neck excluding LG and SG, using peripheral blood tests identified as significantly discriminating the three clinical features. ROC curves were also generated using multiple important factors selected by machine learning to evaluate the change in the area under the ROC curve (AUC). Clinical features were compared between patients divided into two groups by cutoff values obtained from ROC curves.

### 2.4. Machine Learning

Machine learning using random forest was performed by Boruta multivariate analysis (https://notabug.org/mbq/Boruta/). The Boruta feature selection was based on the package “Boruta” in R (version 4.0.2, Vienna University of Economics and Business, Vienna, Austria).

### 2.5. Statistical Analysis

Kruskal–Wallis test and Steel–Dwass test were used to compare clinical features and peripheral blood test data among four groups divided by unbiased clustering analysis and condensed unbiased clustering analysis. Mann–Whitney U test and chi-squared test were used to compare clinical features and peripheral blood test data between two groups divided by the presence or absence of each of the following clinical features: worsened BCVA, enlargement of extraocular muscles, and lesion above neck excluding LG and SG. Mann–Whitney U test and chi-squared test were also used to compare clinical features and peripheral blood test data between two groups divided by the cutoff value obtained from ROC curve. A *p*-value less than 0.05 was considered statistically significant. All analyses were performed using commercial statistical analysis software (BellCurve for Excel, Social Survey Research Information Co. Ltd.; JMP, SAS Institute, Ltd.).

## 3. Results

### 3.1. Patient Demographics and Number of Patients Having Abnormal Peripheral Blood Test

Patient demographics are shown in Table 1. Frequencies of patients with abnormal peripheral blood tests are shown in Table 2. The male/female ratio was also the same, and almost all patients with IgG4-ROD had abnormal serum IgG4 levels.

### 3.2. Unbiased Clustering Analysis Identifies Distinct Groups in IgG4-ROD

**Figure 1:**
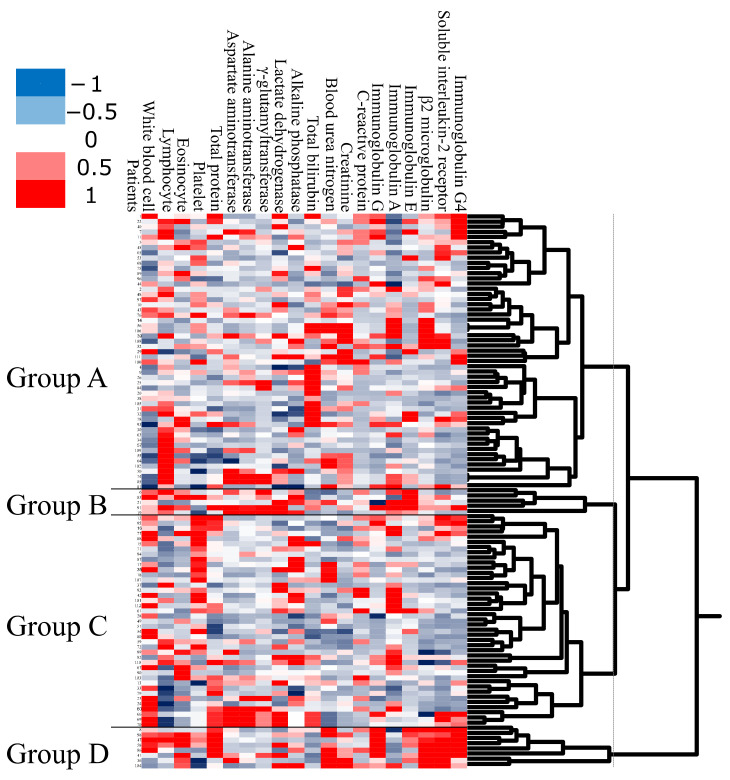
Patients are clustered into four groups by unsupervised hierarchical clustering analysis using the data of 20 peripheral blood tests. Group A, patients with low white blood cell (WBC) count; group B, patients with high serum immunoglobulin E (IgE); group C, patients with high Plt count; and group D, patients with high serum IgG4.

As the first step in the analysis, we applied unbiased clustering analysis to obtain a global overview of laboratory test data (Figure 1). Using unbiased clustering analysis, patients were divided into four groups. Group A was the largest cluster (*n* = 53; 50% of subjects), consisting of patients who had a low WBC count. Group B was the smallest cluster (*n* = 5; 5% of subjects), consisting of patients with high serum IgA and high serum IgE. Group C consisted of 41 subjects (38%) with high platelet count. Group D consisted of 8 subjects (7%) with older onset, high serum IgG, high β2MG, high serum sIL-2R, and high serum IgG4.

We then compared the clinical features between groups A, B, C, and D. The clinical features in the four groups are shown in Table 3. Group D had significantly older onset, higher serum IgG4, and higher frequency of worsened BCVA than the other groups (*p* < 0.001, *p* < 0.001, and *p* = 0.002, respectively). Group B had significantly higher frequency of extraocular muscle enlargement than the other groups (*p* < 0.001). Groups B and D had significantly higher frequency of lesion above neck excluding LG and SG than the other groups (*p* = 0.03). No significant differences were observed among the four groups in other clinical features. These results indicated that peripheral blood test data may be useful for the prediction of the clinical course of IgG4-ROD. However, there were several issues of using 20 peripheral blood tests for such analysis, including cost and whether all the tests are necessary for all patients with IgG4-ROD. Therefore, we performed another clustering analysis to examine whether similar results can be obtained even when the peripheral blood test data were reduced to the minimum.

### 3.3. Condensed Unbiased Clustering Analysis with Reduced Data Identifies Four Distinct Groups in IgG4-ROD 

We performed a condensed unbiased clustering analysis using a smaller number of selected peripheral blood test data. We excluded tests that were deemed unnecessary, costly, or had problems of complexity and reproducibility in clinical use. Peripheral blood tests that showed high frequencies of abnormality in patients with IgG4-ROD were selected for the condensed clustering analysis, as shown in Table 2. The condensed unbiased clustering analysis divided patients into four groups: E, F, G, and H (Figure S1). We then compared the clinical features between these four groups. Group E was the largest cluster (*n* = 41; 38% of subjects), consisting of patients who had a high percent of Eo, high serum IgE, and high serum sIL-2R. Group F was the smallest cluster (*n* = 5; 5% of subjects), consisting of patients who had a high percent of Eo, high serum IgG, high serum IgE, and high serum IgG4. Group G (*n* = 32; 30% of subjects) consisted of patients who had a low WBC count. Group H (*n* = 29; 27% of subjects) consisted of patients who had a low percent of Eo. Clinical features in the four groups divided by the condensed unbiased clustering analysis are shown in Table S1.

Group F had a significantly longer follow-up period, higher serum IgG4, and higher frequency of worsened BCVA than the other groups (*p* = 0.04, *p* < 0.001, and *p* = 0.001, respectively). Group F tended to have higher frequencies of orbital mass, orbital diffuse lesion, optic neuropathy, and visual field defect compared with the other groups (*p* = 0.05, *p* = 0.05, *p* = 0.05, and *p* = 0.05, respectively). On the other hand, the Steel–Dwass test showed that group F had significantly high frequencies of orbital mass, orbital diffuse lesion, optic neuropathy, and visual field defect compared with group G (*p* = 0.02, *p* = 0.02, *p* = 0.02, and *p* = 0.02, respectively). Group E had a significantly higher frequency of extraocular muscle enlargement (*p* = 0.02). Other parameters were not significantly different.

This result also indicated that data of peripheral blood tests may be useful for the prediction of clinical course, especially worsened BCVA and extraocular muscle enlargement, in IgG4-ROD.

### 3.4. Comparison of Clinical Features and Laboratory Findings in Patients Divided into Two Groups Based on Specific Clinical Features

First, we compared the clinical features and laboratory findings between subjects divided into two groups based on extraocular muscle enlargement (Table 4). Patients with extraocular muscle enlargement had significantly higher WBC count, Eo count, CRP, and serum IgE; higher frequencies of lesion excluding LG and SG and lesion above the neck (excluding LG and SG); high frequencies of enlargement of trigeminal nerve and extraocular muscle; a higher frequency of worsened BCVA; and a higher frequency of diplopia (*p* = 0.01, *p* = 0.01, *p* = 0.04, *p* = 0.01, *p* = 0.01, *p* = 0.001, *p* = 0.007, *p* < 0.001, *p* = 0.01, and *p* = 0.007, respectively). Other clinical features and laboratory findings were not significantly difference between the two groups. These results showed that patients with extraocular muscle enlargement had abnormal peripheral blood findings of elevated WBC count, Eo count, CRP, and serum IgE.

We then compared the clinical features and laboratory findings between subjects divided into two groups based on worsened BCVA (Table 5). Patients with worsened BCVA had significantly older onset; higher serum IgG4, WBC count, serum TP, CRP, serum IgG, serum IgA, serum IgE, and β2MG; and higher frequencies of lesion above the neck excluding LG and SG, trigeminal nerve enlargement, extraocular muscle enlargement, orbital mass, orbital diffuse lesion, optic neuropathy, and visual field defect (*p* = 0.01, *p* < 0.001, *p* = 0.003, *p* = 0.004, *p* = 0.004, *p* = 0.02, *p* = 0.03, *p* = 0.04, *p* = 0.002, *p* = 0.02, *p* = 0.01, *p* < 0.001, *p* = 0.006, *p* = 0.006, *p* < 0.001, and *p* < 0.001, respectively).

Other clinical features and laboratory findings were not significantly different between the group with and that without worsened BCVA. These results showed that patients with worsened BCVA had abnormal peripheral blood findings of increased serum IgG4, WBC count, serum TP, CRP, serum IgG, serum IgA, serum IgE, and β2MG.

Next, we compared the clinical features and laboratory findings between subjects divided into two groups based on lesion above the neck excluding LG and SG (Table 6). Patients with lesion above the neck excluding LG and SG had a significantly higher WBC count and higher frequencies of lesion excluding LG and SG, lesion above the neck (excluding LG and SG), lesion below the neck, enlargement of trigeminal nerve, extraocular muscle enlargement, orbital mass, orbital diffuse lesion, worsened BCVA, optic neuropathy, and visual field defect (*p* = 0.01, *p* < 0.001, *p* < 0.001, *p* < 0.001, *p* < 0.001, *p* < 0.001, *p* = 0.001, *p* = 0.001, *p* < 0.001, *p* < 0.001, and *p* < 0.001, respectively).

Other clinical features and laboratory findings were not significantly different between the two groups. These results showed that patients with lesion above neck excluding LG and SG had abnormal peripheral blood finding of an elevated WBC count.

The results of this analysis indicate that routine laboratory findings may be useful for predicting extraocular muscle enlargement, worsened BCVA, and lesion above neck excluding LG and SG in IgG4-ROD.

### 3.5. ROC Curves for Optimal Peripheral Blood Data Predicting Clinical Features

**Figure 2:**
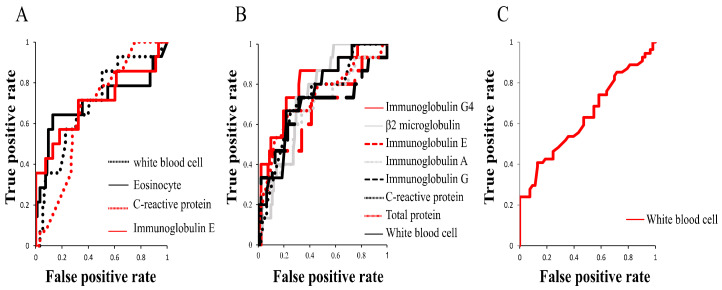
ROC curves for extraocular muscle enlargement, worsened best corrected visual acuity (BCVA), and lesion above the neck excluding LG and SG using significantly different laboratory findings obtained from comparison of laboratory findings in patients with and those without specific clinical features. (**A**). Receiver operating characteristic (ROC) curves for extraocular muscle enlargement using WBC count, Eo count, C-reactive protein (CRP), and IgE. Area under the ROC curve (AUC) of IgE is the largest among the four markers (0.71). Cutoff value of IgE is 425 IU/mL, with the highest odds ratio. (**B**). ROC curves for worsened BCVA using serum IgG4, β2MG, serum IgE, serum IgA, serum IgG, CRP, serum TP, and WBC count. AUC of IgG4 is the largest among the eight markers (0.80). Cutoff value of IgG4 is 713 mg/dL, with the highest odds ratio. (**C**). ROC curve for lesion above the neck excluding LG and SG using peripheral WBC count. AUC of WBC is 0.63. Cutoff value of WBC count is 6700 number/μL.

Having identified the peripheral blood findings that were significantly different between patients divided based on extraocular muscle enlargement, worsened BCVA, or lesion above the neck excluding LG and SG, we plotted ROC curves to investigate which of the laboratory test findings is most suitable for predicting each of the three clinical features (Figure 2 and Table 7). ROC curves for extraocular muscle enlargement using WBC count, Eo count, CRP, and IgE yielded AUCs (95% confidence interval (CI)) of 0.70 (0.55–0.85), 0.70 (0.51–0.90), 0.67 (0.54–0.80), and 0.71 (0.53–0.89), respectively. ROC curve analysis for worsened BCVA using serum IgG4, WBC, TP, CRP, IgG, IgA, IgE, and β2MG yielded AUCs (95% CI) of 0.80 (0.68–0.93), 0.75 (0.62–0.88), 0.73 (0.57–0.89), 0.73 (0.59–0.86), 0.68 (0.50–0.86), 0.67 (0.51–0.83), 0.67 (0.49–0.84), and 0.74 (0.63–0.85), respectively. ROC curves for lesion above the neck excluding LG and SG using WBC count yielded an AUC (95% CI) of 0.63 (0.53–0.74), which was small.

These results indicate that serum IgE is most suitable for the prediction of extraocular muscle enlargement and serum IgG4 is most suitable for the prediction of worsened BCVA, while prediction of lesion above the neck excluding LG and SG using peripheral blood tests may be difficult.

### 3.6. Rank Plot of Feature Importance Using Machine Learning and ROC Curve Using Panel of Tests Selected by Machine Learning

**Figure 3:**
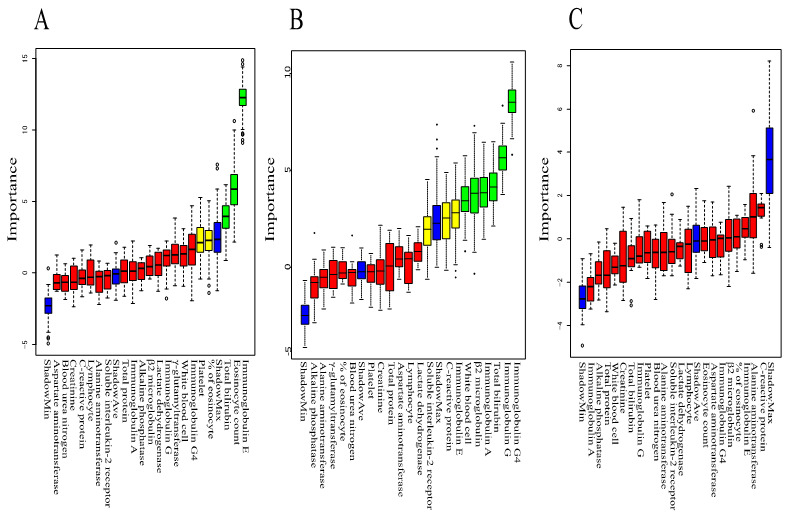
Machine learning analysis using random forest by Boruta multivariate analysis for the prediction of clinical features. Green box denotes a feature confirmed to be important; yellow box denotes a tentative attribute, red box denotes unimportant, and blue box denotes the shadow. (**A**). IgE, Eo count, and T-Bil (three green boxes) are more important for the prediction of extraocular muscle enlargement than other peripheral blood findings. (**B**). WBC, T-Bil, serum IgG, serum IgA, β2-MG, and serum IgG4 (six green boxes) are more important for the prediction of worsened BCVA than other peripheral blood findings. (**C**). All peripheral blood findings are not important for the prediction of lesion above the neck excluding LG and SG. °, * outlier.

We performed machine learning using Boruta to analyze which peripheral blood findings are more important for predicting clinical features such as extraocular muscle enlargement, worsened BCVA, and lesion above the neck excluding LG and SG. The rank plot of feature importance is shown in Figure 3. Serum IgE, Eo count, and T-Bil were more important for extraocular muscle enlargement than other peripheral blood findings. Serum IgG4, serum IgG, T-Bil, serum IgA, β2MG, and WBC count were more important for worsened BCVA than other peripheral blood findings. All peripheral blood findings were not important for lesion above the neck excluding LG and SG. These results indicate that peripheral blood tests are useful for predicting extraocular muscle enlargement and worsened BCVA. On the other hand, the peripheral blood test is not useful for predicting lesion above the neck excluding LG and SG.

**Figure 4:**
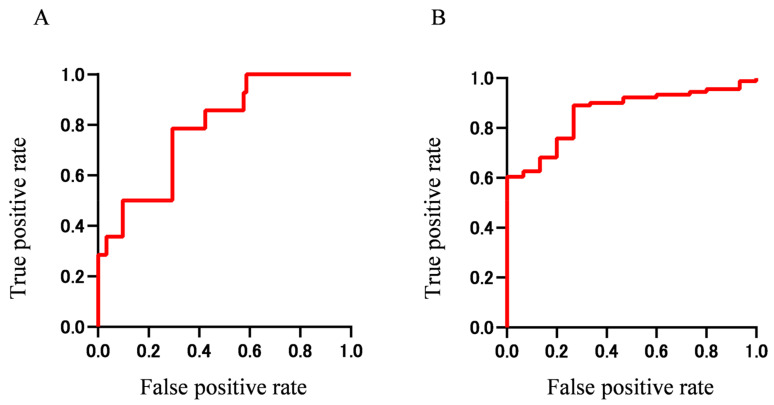
ROC curves for extraocular muscle enlargement (**A**) and worsened BCVA (**B**) using peripheral blood tests identified by Boruta multivariate analysis shown in Figure 3. **A**. AUC is 0.78 by ROC analysis using three green boxes in Figure 3B (IgE, number of Eo, and T-BIL). **B**. AUC is 0.86 by ROC analysis using six green boxes in Figure 3B (WBC, T-BIL, serum IgG, serum IgA, β2-MG, and serum IgG4).

We then generated ROC curves using combinations of the important findings obtained from the Boruta analysis. ROC curves using panels of important findings for extraocular muscle enlargement (IgE, Eo count, and T-Bil) and worsened BCVA (WBC, T-BIL, serum IgG, serum IgA, β2-MG, and serum IgG4) are shown in Figure 4. The AUC was 0.78 (95% CI 0.668–0.904) for extraocular muscle enlargement and 0.86 (95% CI 0.781–0.942) for worsened BCVA, indicting more accurate prediction when using multiple important findings than when using a single finding. These results indicate that extraocular muscle enlargement and worsened BCVA in IgG4-ROD may be predicted with high accuracy using a panel of biomarkers that can be tested routinely in a hospital laboratory.

### 3.7. Difference of Clinical Features in Patients Divided by Concentration of Serum IgG4 or IgE

Finally, we compared clinical features in patients divided by the cut-off serum IgE concentration of 425 IU/mL or cutoff serum IgG4 concentration of 713 mg/dL obtained from ROC analysis (Table 8 and Table 9). Patients with IgE above 425 IU/mL had high serum IgG4 and extraocular muscle enlargement (*p* = 0.01 and *p* = 0.004, respectively). Patients with IgG4 above 713 mg/dL had a high male ratio; older onset; and high frequencies of bilateral LG swelling, lesion excluding LG and SG, lesion above the neck excluding LG and SG, trigeminal nerve enlargement, worsened BCVA, optic neuropathy, visual field defect, and dry eye symptoms (*p* = 0.04, *p* = 0.02, *p* = 0.02, *p* = 0.009, *p* = 0.03, *p* = 0.001, *p* < 0.001, *p* = 0.005, *p* = 0.005, and *p* = 0.04, respectively).

These results indicate that patients with serum IgE above 425 mg/dL are more likely to have extraocular muscle enlargement, while patients with serum IgG4 above 713 mg/dL have a higher probability of lesions involving ocular adnexal tissues and other tissues above the neck, higher frequency of worsened BCVA due to optic neuropathy, and higher frequency of dry eye symptoms.

## 4. Discussion

The Japanese study group of IgG4-ROD reported that the prevalence of IgG4-ROD ranked second among ocular adnexal lymphoproliferative disorders [19]. In that study, among 1014 cases of orbital lymphoproliferative disorders, 404 cases (39.8%) were extranodal mucosa-associated lymphoid tissue (MALT) lymphoma and 219 (21.6%) were IgG4-ROD [19]. Despite this relatively high prevalence, the clinical features of IgG4-ROD remain unclear because only a few studies have analyzed the clinical features of IgG4-ROD in a relatively large sample of over 50 cases [20,21,22]. IgG4-ROD has been reported to cause not only enlargement of ocular adnexal tissues, but also worsening of BCVA due to optic neuropathy [23,24]. Moreover, although systemic steroids are efficacious in all patients with IgG4-RD, some patients cannot be weaned from systemic steroids and require maintenance treatment [22,25,26]. IgG4-RD is a heterogeneous group with diverse clinical phenotypes. Therefore, unbiased clustering analysis is a useful approach to evaluate patient characteristics.

In the first unbiased clustering analysis, groups B and D had a significantly higher frequency of lesion above the neck excluding LG and SG than other groups. Patients with serum IgG4 above 713 mg/dL also had a higher frequency of lesion excluding LG and SG, especially lesion above the neck, than patients with serum IgG4 below 713 mg/dL. In another study of 493 patients with IgG4-RD divided by phenotype, serum IgG4 levels were 316 and 178 mg/dL in pancreato-hepato-biliary disease and aortitis, respectively, while the levels were 445 and 1170 mg/dL in head and neck-limited disease and classic Mikulicz syndrome, respectively.^5^ These results show that IgG4-RD patients with lesion above the neck have higher serum IgG4 than those without above the neck lesion. Moreover, we found that patients with serum IgG4 above 713 mg/dL had higher frequencies of bilateral LG enlargement and dry eye symptoms than patients with serum IgG4 below 713 mg/dL. These findings thus suggest that high serum IgG4 may be a risk factor of multi-organ lesion, particularly lesion above the neck, and dry eye symptoms. On the other hand, patients with lesion above the neck excluding LG and SG had a significantly lower frequency of lesion below the neck in this study. However, Boruta analysis found that none of the peripheral blood test findings including serum IgG4 were important for lesion above neck excluding LG and SG. Even when ROC analysis was conducted for WBC count, the only abnormal laboratory data found in lesion above the neck, the AUC was low (0.63). These results indicate that peripheral blood tests including serum IgG4 are not useful for predicting above the neck lesions excluding LG and SG.

Interestingly, patients with serum IgG4 above 713 mg/dL had a significantly higher frequency of enlargement of trigeminal nerve, but not enlargement of extraocular muscles. Moreover, patients with a high frequency of enlargement of extraocular muscles were not classified into group D or F with high serum IgG4 (above 1000 mg/dL), but into groups B and E with moderate elevation of serum IgG4. Groups B and E consisted of patients who had a high percentage of Eo and high serum IgE. A previous report also indicated that eosinophilia and relatively elevated serum IgG4 were associated with enlargement of extraocular muscles [22]. Moreover, other studies reported that serum IgE was significantly elevated in IgG4-RD [27,28]. These findings indicate the potential importance of Eo and serum IgE in determining severity. IgG4-RD including IgG4-ROD have been shown to be associated with allergy [27]. Analysis using machine learning also revealed that serum IgE, Eo count, and T-BIL were more important for the prediction of extraocular muscle enlargement than other peripheral blood findings in this study. ROC analysis using a combination of three laboratory tests (IgE, Eo count, and T-BIL) selected by machine learning using Boruta had a greater AUC and higher significance compared with that using serum IgG4 alone (AUC 0.80; *p* < 0.001 versus AUC 0.71; *p* = 0.01).

The mechanisms behind the diverse inflammatory markers such as eosinophilia and serum IgE are likely to be complex, but may signify a Th2-driven autoimmune mechanism [29,30]. Therefore, percentage of Eo and serum IgE may be better markers than serum IgG4 for the prediction of enlargement of extraocular muscles. In other words, the presence of variable types of IgG4-ROD may be explained by heterogenous phenotypes including high serum IgG4 without a high percentage of Eo and moderately elevated serum IgG4 with a high percentage of Eo.

Although a case of IgG4-ROD with optic neuropathy was reported [23], optic neuropathy caused by IgG4-ROD is occasionally misdiagnosed as thyroid-associated orbitopathy (TAO) at disease onset, because enlargements of extraocular muscles and LG are also observed in TAO [23,24]. Patients with TAO also show red conjunctiva, eyelid retraction, extraocular muscle enlargement, and increased orbital tissue. Although TAO most frequently affects the inferior rectus muscle, with preferential swelling of the muscle belly, both IgG4-ROD and TAO show similar orbital imaging findings and some clinical features including bilateral disease with a chronic onset, enlargement of extraocular muscles, increased orbital fat, and enlargement of LG [23]. The relationship between TAO and IgG4-ROD has been reported [31,32]. Eight percent of patients with TAO had high serum IgG4 above 135 mg/dL [31]. In IgG4-RD patients, the presence of lesion above the neck may be associated with high serum IgG4 [5]. A case report showed serum IgG4 of 1020 mg/dL in a IgG4-ROD patient with optic neuropathy [23], which is in good agreement with our results. Thus, previous results suggest that IgG4-RD patients with ocular lesion have higher serum IgG4 compared with those without ocular lesion. Our study also found that group D with high serum IgG4 had a significantly higher frequency of orbital lesion than the other groups. Although the ROC curve showed high performance of serum IgG4 alone with AUC of 0.80, a combination of six laboratory tests (WBC, T-BIL, serum IgG, serum IgA, β2-MG, and serum IgG4) obtained from machine learning using Boruta showed a greater AUC (0.86) and higher significance than serum IgG4 alone. Patients with IgG4 above 713 mg/dL, the cutoff level obtained from ROC, had a significantly higher frequency of worsened BCVA. Therefore, these results suggest that serum IgG4 is associated with optic neuropathy owing to orbital lesion, and high serum IgG4 is a risk factor of worsened BCVA. The AUC of serum IgG4 for worsened BCVA was 0.80 in this study (Table 7). Analysis of a large sample of patients with IgG4-ROD may improve the AUC and facilitate interpretation of the model. Therefore, accumulation of more cases in future studies may yield a better AUC and cutoff values.

In this study, WBC, CRP, TP, and β2MG were significantly higher in patients with worsened BCVA than in those with preserved BCVA. One previous study reported higher serum CRP in IgG4-related aortic aneurysms and periaortitis compared with IgG4-related retroperitoneal fibrosis [33]. CRP is a biomarker of inflammatory activity in some rheumatic diseases [34]. Elevated CRP may cause an increase in WBC count. While these results suggest CRP to possibly be a biomarker of IgG4-ROD activity, other reports concluded that serum CRP is not necessarily elevated in IgG4-RD patients with involvement of different organs [35,36]. Therefore, there is controversy over WBC count and CRP as biomarkers of IgG4-ROD. Serum TP is elevated with an increase of serum immunoglobulins, because serum TP consists of serum albumin and immunoglobulins. Hence, serum TP would not be a better biomarker for IgG4-ROD activity than serum IgG4. Serum β2MG increases in other inflammatory diseases [37]. Therefore, IgG4-ROD activity likely causes elevation of serum β2MG. However, Xie et al. [38]. reported that β2MG plays a role in inflammatory diseases as a potential initiator of inflammatory responses. Further study should examine whether β2MG is a useful marker. These inflammatory biomarkers are increasingly being used to elucidate the underlying pathophysiological mechanisms of individual phenotypes, with the hypothesis that each phenotype has a different underlying pathophysiological mechanism.

This study has several strengths and weaknesses. This is the first study to use unsupervised hierarchical cluster analysis to identify a panel of biomarkers in a relatively large sample of patients with IgG4-ROD, despite the rarity of the disease. This study demonstrates the potential of using a panel of commonly available laboratory test data in predicting the prognosis of IgG4-ROD, which will have wide applicability once validated. On the other hand, there are several limitations. First, the retrospective nature of the study may cause selection bias and confounding bias. Thus, the results should be considered exploratory. Second, because all cases were collected from a single tertiary care hospital, there is potential referral bias. Future studies should prospectively validate these laboratory data in independent cohorts and evaluate whether the addition of other markers improves the accuracy of predicting the clinical course such as enlargement of extraocular muscles, worsening of BCVA, and development of extraocular lesions. Future studies are needed to assess the feasibility of using this unbiased cluster analysis in a prospective manner to predict the visual outcome and development of extraocular lesions.

In conclusion, using unbiased clustering analysis, we were able to identify four groups among IgG4-ROD patients, which are associated with different clinical courses, and discover the biomarkers for predicting enlargement of extraocular muscles and worsened BCVA in IgG4-ROD. These phenotypes within IgG4-ROD may in part explain the heterogeneity of this disease. Unbiased clustering analysis reveals that patients with high serum IgE have a high frequency of extraocular muscle enlargement and patients with high serum IgG4 have a high frequency of multi-organ lesion especially lesion above the neck, dry eye symptoms, and worsened BCVA. Surprisingly, although high serum IgG4 alone predicts worsened BCVA among IgG4-ROD patients, using a panel of tests comprising IgG4, IgG, IgA, WBC count, T-BIL, and β2MG improves the accuracy of predicting high risk of worsened BCVA, suggesting that a panel approach may be more effective than serum IgG4 alone, and provides additional evidence for the prevention and management of worsened BCVA in IgG4-ROD. We also noted that these parameters were highly variable among patients. Indeed, for IgG4-ROD patients, data based on serum IgG4 and other markers that can be tested routinely in the hospital laboratory may impact clinical decisions and potentially contribute to the understanding of the mechanisms behind why some patient with IgG4-ROD develop visual disturbance, whereas others do not, and why some patients have extraocular lesions, whereas others do not. A better understanding of this heterogeneity and the link to visual function and extraocular lesions may provide opportunities to conduct personalized or stratified care for IgG4-ROD.

## Figures and Tables

**Table 1:** Patients’ demographics, lesion site, ocular symptom, treatment, and recurrence.

Characteristics	No. (%) or Average (± SD)
Patient demographics	
Gender: male/female	55/52 (51%/49%)
Age (years)	57.3 ± 14.5
Follow-up period (month)	23.0 ± 19.6
Biopsy	79 (74%)
Definite	64 (59%)
Probable	15 (14%)
Possible	28 (26%)
Serum IgG4 (mg/dL)	587.4 ± 575.8
Lesion site	
Lacrimal gland	107 (100%)
Bilateral of lacrimal gland	85 (79%)
Salivary gland	20 (19%)
Lesion other than lacrimal gland and salivary gland	77 (72%)
Higher than the neck	50 (47%)
Lower than the neck	35 (33%)
Enlargement of the trigeminal nerve	14 (13%)
Enlargement of extraocular muscle	14 (13%)
Orbital mass	9 (8%)
Orbital diffuse lesion	9 (8%)
Ocular symptom at the first visit	
Swelling of eyelid	107 (100%)
Worsened best corrected visual acuity	15 (14%)
Optic neuropathy	11 (10%)
Loss of visual field	11 (10%)
Diplopia	14 (13%)
Dry eye	25 (23%)
Treatment and recurrence	
Systemic administration of prednisolone	75 (70%)
Local injection of triamcinolone acetonide	27 (25%)
Recurrence	30 (28%)
Number of recurrence	2.1 ± 1.1

IgG4 = immunoglobulin G4.

**Table 2:** Number of patients who have an abnormal value in the peripheral blood test.

Characteristics	No. (%)	Characteristics	No. (%)
White blood cell	14 (13%)	Blood urea nitrogen	5 (5%)
Lymphocyte	1 (1%)	Creatinine	9 (8%)
Eosinocyte	31 (29%)	C-reactive protein	8 (7%)
Platelet	6 (6%)	Immunoglobulin G	45 (42%)
Total protein	19 (18%)	Immunoglobulin A	11 (10%)
AST	9 (8%)	Immunoglobulin E	69 (64%)
ALT	9 (8%)	β2 microglobulin	41 (38%)
γ-glutamyltransferase	9 (8%)	Soluble interleukin-2 receptor	51 (48%)
Lactate dehydrogenase	15 (14%)	Immunoglobulin G4	95 (89%)
Alkaline phosphatase	3 (3%)		
Total bilirubin	5 (5%)		

AST = aspartate aminotransferase; ALT = alanine aminotransferase.

**Table 3:** Findings in the four groups classified according to clustering analysis.

Characteristics	Group A	Group B	Group C	Group D	*p* Value
Patient demographics					
Gender: male/female	32/21	2/3	15/26	6/2	0.06
Age (years)	59	69.1	39.8	83.9	<0.001
Follow-up period (month)	52.9	46.4	54.7	62.4	0.81
Biopsy	35 (66%)	4 (80%)	31 (76%)	6 (75%)	0.72
Definite	30 (57%)	4 (80%)	24 (59%)	6 (75%)	
Probable	5 (9%)	0 (0%)	10 (24%)	0 (0%)	
Possible	18 (34%)	1 (20%)	7 (17%)	2 (25%)	0.17
Serum IgG4 (mg/dL)	525	579.4	448.6	1717.1	<0.001
Lesion site					
Lacrimal gland	53 (100%)	5 (100%)	41 (100%)	8 (100%)	
Bilateral of lacrimal gland	40 (75%)	4 (80%)	33 (80%)	8 (100%)	0.47
Salivary gland	12 (22%)	1 (20%)	5 (12%)	2 (25%)	0.79
Lesion other than lacrimal gland and salivary gland	38 (72%)	4 (80%)	28 (68%)	7 (88%)	0.71
Higher than the neck	18 (34%)	4 (80%)	22 (54%)	6 (75%)	0.03
Lower than the neck	21 (40%)	1 (20%)	10 (24%)	3 (38%)	0.41
Enlargement of the trigeminal nerve	5 (9%)	2 (40%)	6 (15%)	1 (13%)	0.28
Enlargement of extraocular muscle	4 (8%)	4 (80%)	5 (12%)	1 (13%)	<0.001
Orbital mass	2 (4%)	1 (20%)	4 (10%)	2 (25%)	0.15
Orbital diffuse lesion	2 (4%)	1 (20%)	3 (7%)	3 (38%)	0.01
Ocular symptom at the first visit					
Swelling of eyelid	53 (100%)	5 (100%)	41 (100%)	8 (100%)	
Worsened best corrected visual acuity	5 (9%)	2 (40%)	4 (10%)	4 (50%)	0.002
Optic neuropathy	3 (6%)	1 (20%)	5 (13%)	2 (25%)	0.29
Loss of visual field	3 (6%)	1 (20%)	5 (13%)	2 (25%)	0.29
Diplopia	6 (11%)	1 (20%)	6 (15%)	1 (13%)	0.93
Dry eye	12 (23%)	1 (20%)	9 (22%)	3 (38%)	0.81
Treatment and recurrence					
Systemic administration of prednisolone	36 (68%)	4 (80%)	29 (70%)	6 (75%)	0.93
Local injection of triamcinolone acetonide	13 (25%)	1 (20%)	9 (22%)	4 (50%)	0.41
Recurrence	15 (28%)	1 (20%)	11 (27%)	3 (38%)	0.91
Number of recurrence	2.1 ± 0.9	2	2.3 ± 1.4	1.7 ± 0.5	0.94

**Table 4:** Clinical findings in patients with and those without enlargement of extraocular muscles.

Characteristics	Presence	Absence	*p* Value
Gender: male/female; *n* (%)	7/7	48/45	0.91
Age; years	63.1	56.4	0.12
Follow-up period; months	23.1	20.3	0.75
Biopsy; *n* (%)	8 (57%)	71 (76%)	0.13
Type of IgG4-ROD; *n* (%)			
Definite	6 (43%)	58 (62%)	
Probable	2 (14%)	13 (14%)	
Possible	6 (43%)	22 (24%)	0.29
Serum IgG4; mg/dL	979.8	528.3	0.18
White blood cell; number/μL	7585	6333	0.01
Lymphocyte; %	30.1	29.3	0.86
Eosinocyte; %	7.8	4.2	0.07
Eosinocyte; number/μL	623.2	261.4	0.01
Platelet; number/μL	268.1	240.3	0.26
Total protein; g/dL	8.0	7.6	0.15
Aspartate aminotransferase; U/L	24.3	23.6	0.44
Alanine aminotransferase; U/L	18.1	21.0	0.89
γ-glutamyltransferase; U/L	33.7	31.3	0.79
Lactate dehydrogenase ; U/L	186.9	175.3	0.09
Alkaline phosphatase; U/L	212.1	199.4	0.62
Total bilirubin; mg/dL	0.50	0.67	0.05
Blood urea nitrogen; mg/dL	14.3	13.7	0.49
Creatinine; mg/dL	0.73	0.75	0.23
C-reactive protein; mg/dL	0.22	0.16	0.04
Immunoglobulin G; mg/dL	2087	1733	0.63
Immunoglobulin A; mg/dL	208	205	0.84
Immunoglobulin E; IU/mL	1560	435	0.01
β2 microglobulin; mg/L	1.71	1.50	0.07
Soluble interleukin-2 receptor; U/mL	736	581	0.28
Lesion site; *n* (%)			
Lacrimal gland	14 (100%)	93 (100%)	
Bilateral lacrimal gland	11 (79%)	74 (80%)	0.93
Salivary gland	3 (21%)	17 (18%)	0.77
Lesion other than lacrimal gland and salivary gland	14 (100%)	63 (68%)	0.01
Above the neck	14 (100%)	36 (39%)	0.001
Below the neck	2 (14%)	33 (35%)	0.11
Enlargement of the trigeminal nerve; *n* (%)	5 (36%)	9 (10%)	0.007
Orbital mass; *n* (%)	1 (7%)	8 (9%)	0.85
Orbital diffuse lesion; *n* (%)	1 (7%)	8 (9%)	0.85
Ocular symptom at the first visit; *n* (%)			
Swelling of eyelid	14 (100%)	93 (100%)	
Worsened best corrected visual acuity	5 (36%)	10 (11%)	0.01
Optic neuropathy	3 (21%)	8 (9%)	0.14
Loss of visual field; *n* (%)	3 (21%)	8 (9%)	0.14
Diplopia; *n* (%)	5 (36%)	9 (10%)	0.007
Dry eye; *n* (%)	6 (43%)	19 (20%)	0.06
Treatment; *n* (%)			
Systemic prednisolone	11 (79%)	64 (69%)	0.45
Local injection of triamcinolone acetonide	2 (14%)	25 (27%)	0.31
Recurrence; *n* (%)	3 (21%)	27 (29%)	0.55
Number of recurrence	2.3 ± 0.5	2.1 ± 1.1	0.49

*n* = number of patients; IgG4-ROD = IgG4-related ophthalmic disease.

**Table 5:** Clinical findings in patients with and those without worsened best corrected visual acuity (BCVA).

Characteristics	Worsened BCVA	Non-Worsened BCVA	*p* Value
Gender: male/female; *n* (%)	11/4	44/48	0.07
Age; years	65.2	56.0	0.01
Follow-up period; months	26.1	22.0	0.50
Biopsy; *n* (%)	9 (60%)	70 (76%)	0.19
Type of IgG4-ROD; *n* (%)			
Definite	8 (53%)	56 (61%)	
Probable	1 (7%)	14 (15%)	
Possible	6 (40%)	22 (24%)	0.36
Serum IgG4; mg/dL	1173.8	491.8	<0.001
White blood cell; number/μL	8178.6	6238.0	0.003
Lymphocyte; %	32.0	29.0	0.15
Eosinocyte; %	7.0	4.6	0.25
Eosinocyte; number/μL	449.2	285.8	0.06
Platelet; number/μL	238.3	241.8	0.83
Total protein; g/dL	9.5	7.6	0.004
Aspartate aminotransferase; U/L	24.8	23.4	0.38
Alanine aminotransferase; U/L	20.8	20.6	0.66
γ-glutamyltransferase; U/L	27.9	32.0	0.93
Lactate dehydrogenase ; U/L	165.8	177.3	0.53
Alkaline phosphatase; U/L	222.1	197.1	0.12
Total bilirubin; mg/dL	14.0	0.7	0.08
Blood urea nitrogen; mg/dL	13.6	14.0	0.66
Creatinine; mg/dL	1.4	0.7	0.36
C-reactive protein; mg/dL	0.3	0.2	0.004
Immunoglobulin G; mg/dL	2269.4	1682.70	0.02
Immunoglobulin A; mg/dL	246.7	214.2	0.03
Immunoglobulin E; IU/mL	1062.7	501.0	0.04
β2 microglobulin; mg/L	25.2	1.5	0.002
Soluble interleukin-2 receptor; U/mL	757.7	568.3	0.18
Lesion site; *n* (%)			
Lacrimal gland	15 (100%)	92 (100%)	
Bilateral lacrimal gland	11 (73%)	74 (80%)	0.53
Salivary gland	2 (13%)	18 (20%)	0.57
Lesion other than lacrimal gland and salivary gland	12 (80%)	65 (71%)	0.46
Above the neck	11 (73%)	39 (42%)	0.02
Below the neck	3 (20%)	32 (35%)	0.26
Enlargement of the trigeminal nerve; *n* (%)	5 (33%)	9 (10%)	0.01
Enlargement of extraocular muscle; *n* (%)	7 (47%)	7 (8%)	<0.001
Orbital mass; *n* (%)	5 (35%)	4 (4%)	0.006
Orbital diffuse lesion; *n* (%)	5 (35%)	4 (4%)	0.006
Ocular symptom at the first visit; *n* (%)			
Swelling of eyelid	15 (100%)	92 (100%)	
Optic neuropathy	10 (67%)	1 (1%)	<0.001
Loss of visual field; *n* (%)	10 (67%)	1 (1%)	<0.001
Diplopia; *n* (%)	4 (27%)	10 (11%)	0.09
Dry eye; *n* (%)	6 (40%)	19 (21%)	0.10
Treatment; *n* (%)			
Systemic prednisolone	13 (87%)	62 (67%)	0.13
Local injection of triamcinolone acetonide	3 (20%)	24 (26%)	0.62
Recurrence; *n* (%)	3 (20%)	27 (29%)	0.46
Number of recurrence	2.0 ± 0.8	2.1 ± 1.1	0.87

*n* = number of patients.

**Table 6:** Clinical findings in patients with and those without lesion above the neck excluding lacrimal gland (LG) and salivary gland (SG).

Characteristics	Presence	Absence	*p* Value
Gender: male/female; *n* (%)	30/20	25/32	0.09
Age; years	59.7	54.9	0.06
Follow-up period; months	20.6	19.8	0.40
Biopsy; *n* (%)	33 (66%)	46 (80%)	0.08
Type of IgG4-ROD; *n* (%)			
Definite	25 (50%)	39 (68%)	
Probable	8 (16%)	7 (12%)	
Possible	17 (34%)	11 (19%)	0.13
Serum IgG4; mg/dL	692.4	480.4	0.26
White blood cell; number/μL	6898	6000	0.01
Lymphocyte; %	28.5	30.3	0.37
Eosinocyte; %	5.4	4.0	0.28
Eosinocyte; number/μL	390.6	225.4	0.07
Platelet; number/μL	236.4	251.6	0.09
Total protein; g/dL	7.8	7.6	0.34
Aspartate aminotransferase; U/L	24.0	23.3	0.64
Alanine aminotransferase; U/L	21.2	20.1	0.68
γ-glutamyltransferase; U/L	35.6	27.6	0.88
Lactate dehydrogenase ; U/L	180.5	173.1	0.36
Alkaline phosphatase; U/L	201.6	200.7	0.99
Total bilirubin; mg/dL	0.62	0.67	0.18
Blood urea nitrogen; mg/dL	14.9	12.6	0.05
Creatinine; mg/dL	0.78	0.72	0.42
C-reactive protein; mg/dL	0.16	0.18	0.38
Immunoglobulin G; mg/dL	1879.9	1676.8	0.87
Immunoglobulin A; mg/dL	197.4	213.5	0.38
Immunoglobulin E; IU/mL	668.9	493.5	0.61
β2 microglobulin; mg/L	1.57	1.46	0.47
Soluble interleukin-2 receptor; U/mL	649.0	553.1	0.48
Lesion site; *n* (%)			
Lacrimal gland	50 (100%)	57 (100%)	
Bilateral lacrimal gland	36 (72%)	49 (86%)	0.07
Salivary gland	8 (16%)	12 (21%)	0.50
Lesion other than lacrimal gland and salivary gland	45 (90%)	32 (56%)	<0.001
Below the neck	6 (12%)	29 (51%)	<0.001
Enlargement of the trigeminal nerve; *n* (%)	14 (28%)	0 (0%)	<0.001
Enlargement of extraocular muscle; *n* (%)	14 (28%)	0 (0%)	<0.001
Orbital mass; *n* (%)	9 (18%)	0 (0%)	0.001
Orbital diffuse lesion; *n* (%)	9 (18%)	0 (0%)	0.001
Ocular symptom at the first visit; *n* (%)			
Swelling of eyelid	50 (100%)	57 (100%)	
Worsened best corrected visual acuity	14 (28%)	1 (2%)	<0.001
Optic neuropathy	11 (22%)	0	<0.001
Loss of visual field; *n* (%)	11 (22%)	0	<0.001
Diplopia; *n* (%)	10 (20%)	4 (7%)	0.09
Dry eye; *n* (%)	13 (26%)	12 (21%)	0.54
Treatment; *n* (%)			
Systemic prednisolone	37 (74%)	38 (67%)	0.41
Local injection of triamcinolone acetonide	9 (18%)	18 (32%)	0.10
Recurrence; *n* (%)	14 (28%)	16 (28%)	0.99
Number of recurrence	2.3 ± 1.0	1.9 ± 1.1	0.25

*n* = number of patients.

**Table 7:** Cut-off values obtained from receiver operating characteristic (ROC) curve.

Markers	AUC	Odds Ratio	Cut Off Value
Extraocular muscle enlargement			
White blood cell	0.70	4.0	7000/μL
Eosinocyte	0.70	12.2	441/μL
C-reactive protein	0.67	4.8	0.14 mg/dL
Immunoglobulin E	0.71	5.3	425 IU/mL
Worsened best corrected visual acuity			
Immunoglobulin G4	0.80	9.9	713 mg/dL
White blood cell	0.75	7.2	7700/μL
Total protein	0.73	6.4	7.9 g/dL
C-reactive protein	0.73	6.0	0.15 mg/dL
Immunoglobulin G	0.68	6.0	1760 mg/dL
Immunoglobulin A	0.67	3.9	177 mg/dL
Immunoglobulin E	0.67	3.9	348 IU/mL
β2 microglobulin.	0.74	6.6	1.62 mg/L
Lesion above neck			
White blood cell	0.63	4.5	7700/μL

AUC = area under ROC curve.

**Table 8:** Clinical findings in patients stratified by serum IgE concentration of 425 IU/ml.

Characteristics	Below 425 IU/mL	Above 425 IU/mL	*p* Value
Gender: male/female; *n* (%)	30/37	25/15	0.07
Age; years	56.5	58.6	0.38
Follow-up period; months	23.1	21.7	0.81
Biopsy; *n* (%)	49 (73%)	30 (75%)	0.83
Type of IgG4-ROD; *n* (%)			
Definite	36 (54%)	28 (70%)	
Probable	13 (19%)	2 (5%)	
Possible	18 (27%)	10 (21%)	0.08
Serum IgG4; mg/dL	448.3	820.3	0.01
Lesion site; *n* (%)	181.8	1252.5	<0.001
Lacrimal gland	67 (100%)	40 (100%)	
Bilateral lacrimal gland	52 (78%)	33 (83%)	0.54
Salivary gland	11 (16%)	9 (23%)	0.43
Lesion other than lacrimal gland and salivary gland	46 (69%)	33 (83%)	0.11
Above the neck	30 (45%)	20 (50%)	0.94
Below the neck	21 (31%)	14 (35%)	0.69
Enlargement of the trigeminal nerve; *n* (%)	6 (9%)	8 (20%)	0.10
Enlargement of extraocular muscle; *n* (%)	4 (6%)	10 (25%)	0.004
Orbital mass; *n* (%)	6 (9%)	3 (8%)	0.79
Orbital diffuse lesion; *n* (%)	5 (7%)	4 (10%)	0.64
Ocular symptom at the first visit; *n* (%)			
Swelling of eyelid	30 (100%)	77 (100%)	
Worsened best corrected visual acuity	7 (10%)	8 (20%)	0.17
Optic neuropathy	7 (10%)	4 (10%)	0.94
Loss of visual field; *n* (%)	7 (10%)	4 (10%)	0.94
Diplopia; *n* (%)	9 (13%)	5 (13%)	0.89
Dry eye; *n* (%)	13 (19%)	12 (30%)	0.12
Treatment; *n* (%)			
Systemic prednisolone	44 (66%)	31 (78%)	0.19
Local injection of triamcinolone acetonide	15 (22%)	12 (30%)	0.38
Recurrence; *n* (%)	21 (31%)	9 (23%)	0.32
Number of recurrence	2.1 ± 1.2	2.1 ± 0.7	0.68

*n* = number of patients.

**Table 9:** Clinical findings in patients stratified by serum IgG4 concentration of 713 mg/dL.

Characteristics	Below 713 mg/dL	Above 713 mg/dL	*p* Value
Gender: male/female; *n* (%)	35/42	20/10	0.04
Age; years	55.3	62.5	0.02
Follow-up period; months	20.8	26.9	0.34
Biopsy; *n* (%)	56 (73%)	23 (77%)	0.67
Type of IgG4-ROD; *n* (%)			
Definite	42 (55%)	22 (73%)	
Probable	14 (18%)	1 (3%)	
Possible	21 (27%)	7 (23%)	0.09
Serum IgG4; mg/dL	293.3	1342.1	<0.001
Lesion site; *n* (%)			
Lacrimal gland	77 (100%)	30 (100%)	
Bilateral lacrimal gland	57 (74%)	28 (93%)	0.02
Salivary gland	12 (16%)	8 (27%)	0.18
Lesion other than lacrimal gland and salivary gland	50 (65%)	27 (90%)	0.009
Above the neck	31 (40%)	19 (63%)	0.03
Below the neck	22 (29%)	13 (43%)	0.14
Enlargement of the trigeminal nerve; *n* (%)	5 (6%)	9 (30%)	0.001
Enlargement of extraocular muscle; *n* (%)	7 (9%)	7 (23%)	0.05
Orbital mass; *n* (%)	5 (6%)	4 (13%)	0.26
Orbital diffuse lesion; *n* (%)	5 (6%)	4 (13%)	0.26
Ocular symptom at the first visit; *n* (%)			
Swelling of eyelid	77 (100%)	30 (100%)	
Worsened best corrected visual acuity	4 (5%)	11 (37%)	<0.001
Optic neuropathy	4 (5%)	7 (23%)	0.005
Loss of visual field; *n* (%)	4 (5%)	7 (23%)	0.005
Diplopia; *n* (%)	8 (10%)	6 (20%)	0.18
Dry eye; *n* (%)	14 (18%)	11 (37%)	0.04
Treatment; *n* (%)			
Systemic prednisolone	51 (66%)	24 (80%)	0.16
Local injection of triamcinolone acetonide	18 (23%)	9 (30%)	0.48
Recurrence; *n* (%)	24 (31%)	6 (20%)	0.25
Number of recurrence	2.1 ± 1.2	2.2 ± 0.7	0.62

*n* = number of patients.

## Supplementary Materials

The following are available online at https://www.mdpi.com/2077-0383/9/12/4084/s1, Figure S1: The patients clustered into four groups by condensed unbiased clustering analysis using 10 peripheral blood findings; Table S1: Clinical findings in four groups classified according to new clustering analysis.
