# Supplementary material for: Identification of Markers Predicting Clinical Course in Patients with IgG4-Related Ophthalmic Disease by Unbiased Clustering Analysis
Source: J Clin Med. 2020 Dec 17;9(12):4084. doi: 10.3390/jcm9124084 (PMC7766793; doi:10.3390/jcm9124084)
Supplement: Supplementary file 1 [file jcm-09-04084-s001.pdf]

Supplementary Materials

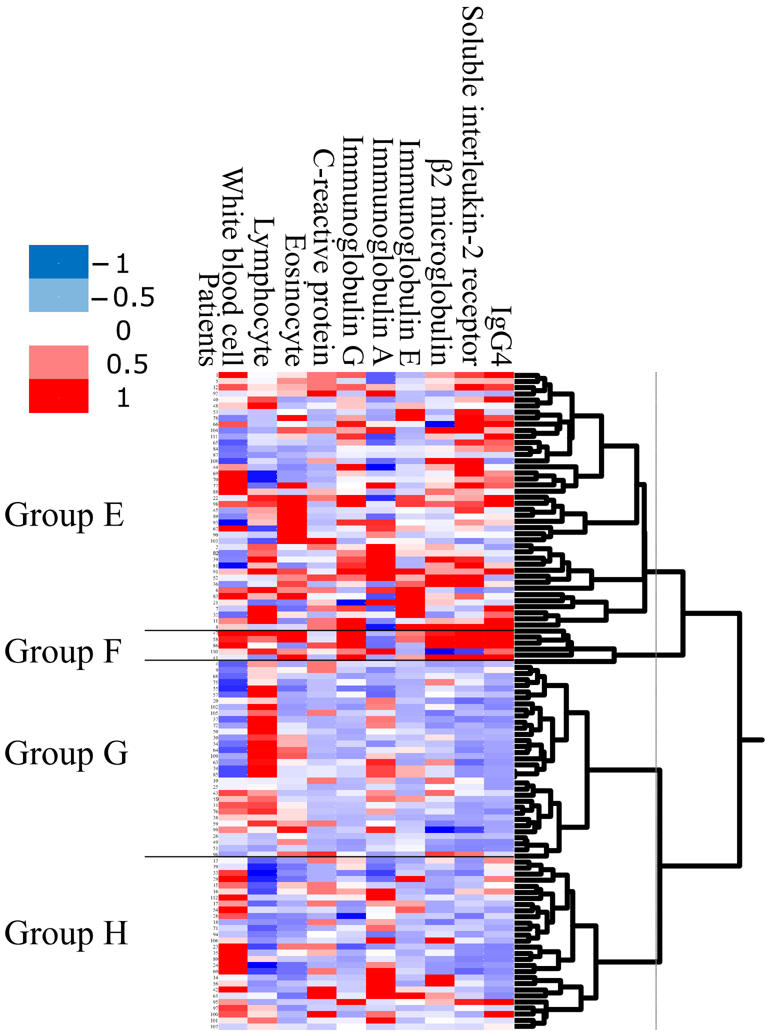

Figure S1. The patients clustered into four groups by condensed unbiased clustering analysis using 10 peripheral blood findings. Group E, patients with high serum immunoglobulin E (IgE); group F, patients with high serum immunoglobulin G4 (IgG4); group G, patients with low white blood cell WBC count; and group H, patients with low percent of eosinocyte.

Table S1. Clinical findings in four groups classified according to new clustering analysis.

Characteristics	Group E	Group F	Group G	Group H	p value
Patient demographics					
Gender : male/female	27/14	2/3	14/18	12/17	0.13
Age (years)	57.8	74.2	52.1	47.2	0.23
Follow-up period (month)	20.5	32.8	18.4	28.2	0.04
Biopsy	29 (71%)	5 (100%)	21 (66%)	24 (83%)	0.23
Definite	24 (59%)	4 (80%)	16 (50%)	20 (69%)	
Probable	5 (12%)	1 (20%)	5 (16%)	4 (14%)	
Possible	12 (29%)	0 (0%)	11 (34%)	5 (17%)	0.58
Serum IgG4 (mg/dL)	771	1713	203	558	<0.001
Lesion site					
LG	41 (100%)	5 (100%)	32 (100%)	29 (100%)	
Bilateral of LG	33 (80%)	5 (20%)	22 (69%)	25 (86%)	0.23
SG	10 (24%)	1 (20%)	6 (19%)	3 (10%)	0.53
Lesion other than LG and SG	30 (73%)	3 (60%)	24 (75%)	20 (69%)	0.88
Higher than the neck	20 (49%)	2 (40%)	15 (47%)	13 (45%)	0.98
Lower than the neck	15 (37%)	3 (60%)	10 (31%)	7 (24%)	0.40
Enlargement of the trigeminal nerve	8 (20%)	1 (20%)	0 (0%)	5 (17%)	0.09
Enlargement of extraocular muscle	10 (24%)	0 (0%)	0 (0%)	4 (14%)	0.02
Orbital mass	3 (7%)	2 (40%)	0 (0%)	3 (10%)	0.05
Orbital diffuse lesion	3 (7%)	2 (40%)	1 (3%)	3 (10%)	0.05
Ocular symptom at the first visit					
Swelling of eyelid	41 (100%)	5 (100%)	32 (100%)	29 (100%)	
Decreasing of BCVA	9 (22%)	3 (60%)	1 (3%)	2 (7%)	0.001
Optic neuropathy	6 (15%)	2 (40%)	1 (3%)	2 (7%)	0.05
Loss of visual field	6 (15%)	2 (40%)	1 (3%)	2 (7%)	0.05
Diplopia	7 (17%)	1 (20%)	1 (3%)	5 (17%)	0.26
Dry eye	10 (24%)	3 (60%)	4 (13%)	8 (28%)	0.11
Treatment and recurrence					
Systemic administration of PSL	30 (73%)	4 (80%)	18 (56%)	23 (79%)	0.21
Local injection of triamcinolone acetonide	10 (24%)	3 (60%)	6 (19%)	8 (28%)	0.26
Recurrence	9 (22%)	2 (40%)	9 (28%)	10 (34%)	0.64
Number of recurrence	1.7 ± 0.5	1.5 ± 0.5	2.1 ± 0.9	2.6 ± 1.4	0.42

LG = lacrimal gland; SG = salivary gland; BCVA = best corrected visual acuity; PSL = prednisolone.
